# Dynamic sagittal balance evaluated by 3-dimentional gait analysis in patients with degenerative lumbar kyphosis

**DOI:** 10.1186/1748-7161-10-S1-P11

**Published:** 2015-01-19

**Authors:** Yo Shiba, Hiroshi Taneichi, Satoshi Inami, Hiroshi Moridaira, Daisaku Takeuchi, Makoto Ohe, Yutaka Nohara

**Affiliations:** 1 Dokkyo Medical University, Japan

## Purpose

To analyze dynamic sagittal global balance (SGB) in patients with degenerative lumbar kyphoscoliosis (DLKS) and to reveal difference between static and dynamic SGB.

## Method

Twenty-six patients with DLKS underwent gait analysis using 3-dimentional motion analysis system (3D-MAS). Two reflection markers were attached to the skin surface on C7 and S1 spinous processes in all patients.

Then they walked on treadmill at self-selected walking speed, and 3-dementional location of these surface makers (C7.S1) were recorded by synchronized 4 cameras that were placed separately. The angles between vertical axis and the line C7-S1 (dynamic-trunk angle: D-TA) and horizontally projected distance of the line C7-S1 (dynamic sagittal vertical axis; D-SVA) were continuously recorded during walking. D-TA and D-SVA were compared to static trunk angle (S-TA) and static SVA(S-SVA) that were obtained regular standing full-length lateral X-ray.

## Result

The patients ceased their walking on the treadmill at 4.0 minutes on the average due to back pain or sever spinal fatigue. A mean D-TA was 27.4degrees at the beginning of walking and/30.1degees at the end of walking. Whereas, D-SVA was 16.3cm at the beginning of walking and 22.1cm at the end of walking on the average. A mean change of D-TA and D-SVA between the beginning and the end of walking were 5.8 degrees and 4.7cm, respectively. On the other hand, an average S-TA and S-SVA were 17.6 degrees and, 12.8cm, respectively. D-SVA was significantly greater than S-SVA not only at the end of walking but the beginning of walking (p=0.06,p<0.001). D-TA was significantly larger than S-TA as well (p<0.001,p<0.001).

## Conclusion

In this study, we revealed that dynamic SGB during walking was significantly worse than static SGB in the patients with DLKS. Compensated static SGB at standing was no longer maintained after start of walking because compensatory mechanisms such as retroversion of the pelvis did not work. Gait analysis using 3D-MAS gave us to useful about real dynamic balance that cannot be detected SVA.

